# Kynurenine serves as useful biomarker in acute, Long- and Post-COVID-19 diagnostics

**DOI:** 10.3389/fimmu.2022.1004545

**Published:** 2022-09-23

**Authors:** Daniel Alexander Bizjak, Manfred Stangl, Nikolaus Börner, Florian Bösch, Joachim Durner, Gergana Drunin, Jasmine-Leonike Buhl, Dietmar Abendroth

**Affiliations:** ^1^ Division of Sports and Rehabilitation Medicine, Ulm University Hospital, Ulm, Germany; ^2^ Division of General, Visceral and Transplant Surgery, Hospital Großhadern, Ludwig-Maximilians-University, Munich, Germany; ^3^ Neurology Department, Special Medical Clinic Ichenhausen, Ichenhausen, Germany; ^4^ Division of Surgery, Ulm University Hospital, Ulm, Germany

**Keywords:** kynurenine reference values, inflammation diagnostics, COVID-19 monitoring, Long-COVID biomarkers, innate immunity

## Abstract

**Introduction:**

In patients with SARS-CoV-2, innate immunity is playing a central role, depicted by hyperinflammation and longer lasting inflammatory response. Reliable inflammatory markers that cover both acute and long-lasting COVID-19 monitoring are still lacking. Thus, we investigated one specific inflammatory marker involved as one key player of the immune system, kynurenine (Kyn), and its use for diagnosis/detection of the Long-/Post-COVID syndrome in comparison to currently used markers in both serum and saliva samples.

**Material and methods:**

The study compromised in total 151 inpatients with a SARS-CoV-2 infection hospitalized between 03/2020 and 09/2021. The group NC (normal controls) included blood bank donors (n=302, 144f/158m, mean age 47.1 ± 18.3 years (range 18-75)). Two further groups were generated based on Group A (n=85, 27f/58m, mean age 63.1 ± 18.3 years (range 19-90), acute admission to the hospital) and Group B (n=66, 22f/44m, mean age 66.6 ± 17.6 years (range 17-90), admitted either for weaning or for rehabilitation period due to Long-COVID symptoms/syndrome). Plasma concentrations of Kyn, C-Reactive Protein (CRP) and interleukin-6 (IL-6) were measured on admission. In Group B we determined Kyn 4 weeks after the negative PCR-test. In a subset of patients (n=11) concentrations of Kyn and CRP were measured in sera and saliva two, three and four months after dismission. We identified 12 patients with Post-COVID symptoms >20 weeks with still significant elevated Kyn-levels.

**Results:**

Mean values for NC used as reference were 2.79 ± 0.61 µM, range 1.2-4.1 µM. On admission, patients showed significantly higher concentrations of Kyn compared to NC (p-values < 0.001). Kyn significantly correlated with IL-6 peak-values (r=0.411; p-values <0.001) and CRP (r=0.488, p-values<0.001). Kyn values in Group B (Long-/Post-COVID) showed still significant higher values (8.77 ± 1.72 µM, range 5.5-16.6 µM), whereas CRP values in Group B were in the normal range.

**Conclusion:**

Serum and saliva Kyn are reflecting the acute and long-term pathophysiology of the SARS-CoV-2 disease concerning the innate immune response and thus may serve a useful biomarker for diagnosis and monitoring both Long- and Post-COVID syndrome and its therapy.

## Introduction

The mutual reaction of host defense against pathogens generally exhibit an initial tissue injury mediated by various generated pathogen-associated molecular patterns (PAMPs) and by any injurious nonpathogenic factors that includes the generation and appearance of damage-associated molecular patterns (DAMPs) (1).

There is evidence that in the center of tissue injury, reactive oxygen species (ROS) play a dominant role and that its origin (e.g., infectious, toxic, physical, or other injurious events) has only a minor effect (2). Oral gingival epithelium as well as the airway epithelium are predisposed as a sentinel system to detect pathogens and nonpathogenic agents and to initiate a host innate defense response (3–5).

The innate immune responses and respective involved cell types play a vital role in the origin of clinical symptoms and severity of COVID-19 disease. This assumption is in agreement with previous studies on the SARS-CoV, which is the closest relative to SARS-CoV-2, and that predominantly infects airway and alveolar epithelial cells, vascular endothelial cells, and macrophages (6). It has been demonstrated that SARS-CoV can influence and trigger various innate recognition and response pathways (6).

The prevailing evidence suggests that patients with severe COVID-19 seem to have an overreaction of the innate immune system demonstrating exacerbated levels of inflammation caused by a so-called “cytokine storm” (6). Although COVID-19 has been closely examined in the last two years regarding acute and long-term mental and physiological health consequences, the versatile mechanisms that underpin COVID-19 are still intensely studied with regard to possible symptoms and health outcome (6, 7).

Inside the tryptophan metabolism, the kynurenine pathway (KP) plays a critical role in generating cellular energy in the form of nicotinamide adenine dinucleotide (NAD^+^). Especially during an immune response, energy requirements are substantially increased and the KP acts a key regulator of the immune system (8). This key regulator is of utmost importance especially in the line of first defense in the innate immune activation (9).

Kynurenine is known to signal through the aryl hydrocarbon receptor (Ahr) with the possibility for modulation of ROS levels (10). The Ahr promoter region contains several sites for NF-kB binding, indicating that inflammation is a key factor modulating Ahr expression. Furthermore, kynurenine activation of Ahr stimulate expression of the enzyme Indoleamine 2,3-dioxygenase 1 (IDO1), which generates kynurenine by degrading tryptophan (11). On the one hand, this positive feedback loop may link inflammation with ROS production, whereas on the other hand, the antioxidant Nrf2 can be stimulated by Ahr, and Nrf2 can itself activate Ahr expression. The balance between pro- and anti-oxidative functions of Ahr mediated by kynurenine may therefore regulate healthy versus unhealthy aging in different tissues and organ systems (11).

Kynurenine is metabolized by IDO-1 in the brain. Prolonged exposure by chemokines due to increased kynurenine levels may result in long-term brain impairment. Kynurenine metabolites itself are producing pro-oxidative and pro-inflammatory effects, resulting in impairment of cognitive function, enhanced oxidative stress and decreased brain-derived neurotrophic factor. The place of action is located in the microglia cells, responsible as innate immune cells (9, 12).

The mechanistic pathways especially in the brain through which the kynurenines interact with these systems are well known, and the subsequent inflammation and inflammatory events induced by e.g., virus-driven diseases can negatively affect emotion, cognition, pain, metabolic function, and aging (8). In doing so, abnormal concentrations or a disbalance of kynurenine metabolites have the potential for increasing the risk of developing psychiatric disorders (13, 14).

The currently most prominent example of the virus-driven activation and not successfully downregulated innate immune response together or alone with a cytokine storm event is the so-called Long-COVID syndrome in adults, or the PIMS (pediatric inflammatory multiorgan syndrome) in children (15). These syndromes include long-term health consequences including impaired healing in the brain (depression), in the lung (fibrosis), in the cardiovascular system (loss of heart function, endothelial reaction) and in the kidney (loss of renal function). The disease exhibits symptoms like severe pneumonia, associated to a severe inflammatory reaction including high C-reactive protein (CRP) and interleukin-6 (IL-6) levels, low albumin and eosinophils, but high sedimentation rate and lymphopenia. Hospitalized individuals also have increased lactate dehydrogenase (LDH), a marker of cellular death, often associated with altered coagulation (16, 17). With more than 90% accuracy, high concentrations of high-sensitivity CRP and LDH as well as a low lymphocyte count can predict mortality of individual patients more than 10 days in advance (18). Several meta-analyses also associated IL-6 levels with the severity of COVID-19 syndrome (19–21).

The Long-COVID syndrome is a longer lasting subclinical inflammation in different parts of the body yet, leading in some individuals to chronic manifestations called Post-COVID syndrome (> 12 weeks), but the diversity of symptoms and individual disease progression indicates that there is no main clinical denominator of biomarkers so far (22–24). The duration and kind of predictive forecasting of the COVID-19 infection by measuring this sustained subclinical inflammation might thus be a valuable asset for clinical diagnostics.

This asset might be the aforementioned aromatic amino acid kynurenine. It has been observed that in some cases, SARS-CoV-2 elicits a reaction like the cytokine storm syndrome seen in sepsis. During sepsis, dendritic cells showing an “overproduction”of IDO, leading to a powerful counter-regulatory, anti-inflammatory reaction characterized by apoptosis of immune effector cell and cellular (especially T cell) exhaustion. The resulting consequence is often seen by immunosuppression and increased susceptibility to secondary infections (25–28). Recently, a meta-analysis showed that the kynurenine pathway is extremely active in acute COVID-19, accompanied by reduced tryptophan and elevated kynurenine, and much more active in severe COVID-19 patients compared to mild or moderate patients (29).

All these patterns are related to the tryptophan-metabolism and its central molecule, kynurenine. The ongoing elevation of the tryptophan metabolism downstream including kynurenine and kynurenic acid is thus of utmost importance.

The main purpose of this proof-of-concept study was 1) to examine if kynurenine is able to depict the inflammatory situation during the acute phase of the disease and 2) if so, if there will be the theoretically developed prognosis of an ongoing subclinical inflammatory situation in patients with the Long-/Post-COVID syndrome.

## Material and methods

### Study population

The study compromised in total 151 inpatients with a SARS-CoV-2 infection hospitalized between 03/2020 and 09/2021 in LMU Munich (Großhadern). The group NC (normal controls) included blood bank donors (n=302, 144f/158m, mean age 47.1 ± 18.3 years (range 18-75)). Detailed NC characteristic can be found in Kaden et al. (2015) and Abendroth et al. (2014) (30, 31).

Two further groups were generated based on acute admission at the hospital:

Group **A** (n=85, 27 f/58 m, mean age 63.1 ± 18.3 years (range 19-90), admitted LMU Großhadern) was treated either on the infection ward (n=67) or the Intensive care Unit (ICU) (n=18), whereas Group **B** was admitted either for weaning or for rehabilitation period due to Long-COVID symptoms/syndrome (n= 66, 22 f/44 m, mean age 66.6 ± 17.6 years (range 17-90), admitted Rehabilitation Hospital Ichenhausen). Plasma concentrations of kynurenine, CRP and interleukin-6 (IL-6) were measured on admission (**Table 1**).

**Table 1:** Demographic and biochemical data of group NC, A, B.

Group	NC, Normal Controls n = 302	A, Acute COVID-19 n = 85	B, Long-COVID-19 n = 66	p-value
Age (years)	48.3 ± 18.3	63.1 ± 16.5	66.6 ± 17.6	B-C
	(Range 18-75)	(Range 19-90)	(Range 17-90)	n.s.
Gender (f/m)	144/158	27/58	22/44	
ICU (n/%)	n.a.	18/22%	6/9%	
Ventilation (days)	n.a.	12 (3 – 131 days)	Weaning	n.a.
**Biochemical Parameter**				
Kynurenine (µM)	2.79 ± 0.61	10.18 ± 8.88	9.01 ± 3.62	NC vs. A/B p<0.001
				A vs. B p=0.182
CRP (mg/L)	< 5	69.2 ± 14.9	Admission: Month 2: 4.14 ± 2.1 Month 3: 1.88 ± 0.8 > Month 5: 1.28 ± 0.5	n.a.
IL-6 pg/ml (peak)	<1.0	58.8 ± 17.4	n.d.	n.a.

All three groups were comparable concerning age and gender distribution. There was a significant difference concerning kynurenine between the normal controls and patients with COVID-19 infections or with Long-COVID syndrome. All data are given as mean ± standard deviation if not otherwise stated. The respective range is presented in parentheses. (ICU, intensive care unit; n.a., not applicable; n.d., not done; n.s., not significant).

**Figure 1:**
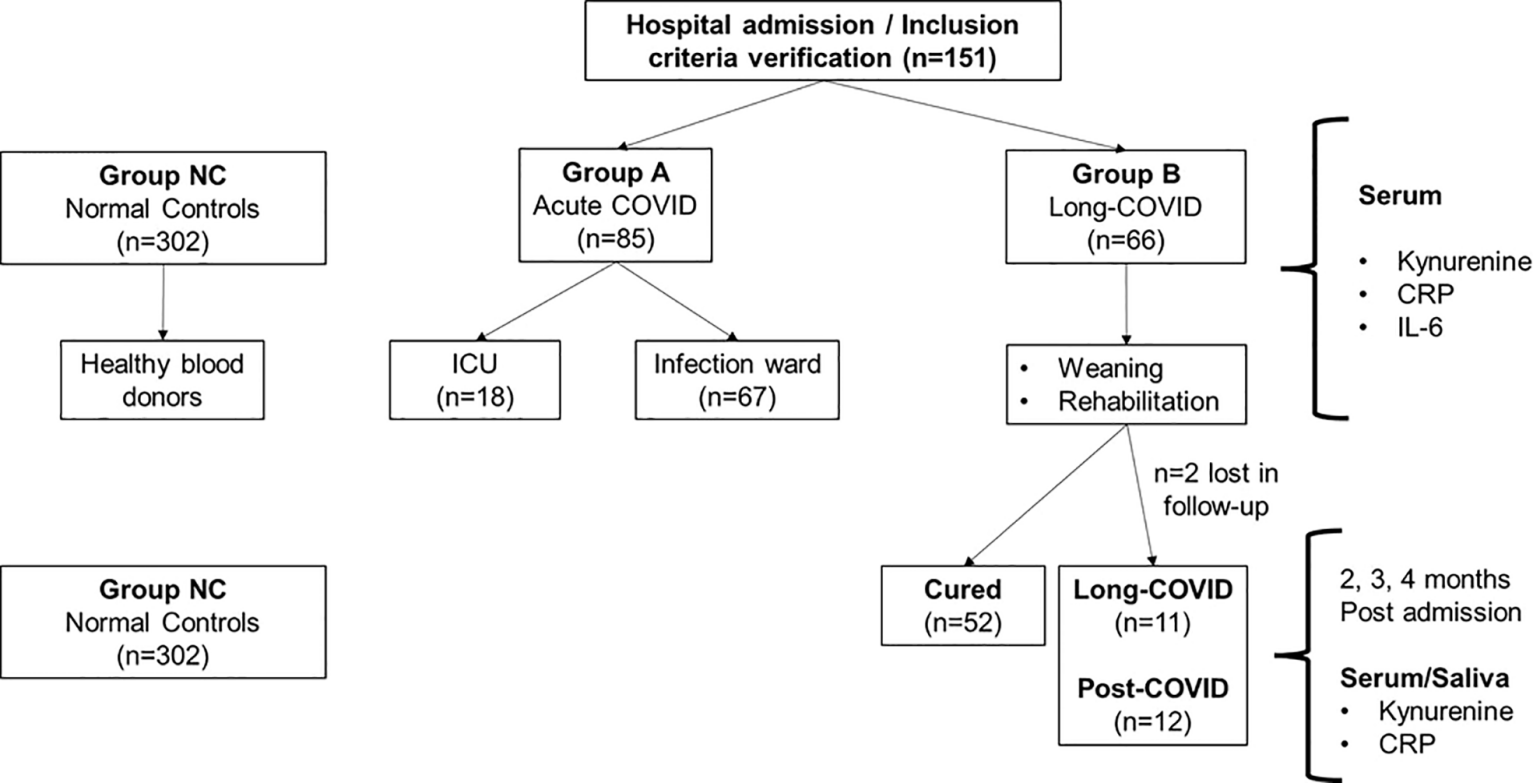
Overview of the different study populations and the respective experimental biomarkers. Group NC (normal controls) consisted of healthy blood donors (30, 31) where kynurenine concentrations were measured and taken as reference values for the Sars-CoV-2 infected individuals (Group A and Group B). ICU = Intensive Care Unit; CRP = C-Reactive Protein; IL-6 = Interleukin 6.

In Group **B** kynurenine concentrations were determined 4 weeks after the negative PCR-test. In a subset of patients (n=11) concentrations of kynurenine and CRP were measured in sera and saliva 2, 3 and 4 months after dismission in a pilot-like sub study. The same protocol was used for saliva measurements withdrawn for PCR-Analysis for detection of COVID-19 antibodies (n=11). **Figure 1** illustrates the different groups with the experimental determined biomarkers at the respective time points.

As has been shown earlier, there exists a linear correlation (r^2=^ 0.902) between the measurement of kynurenine in serum and in saliva (24).

### Ethics

The study was released and signed by the ethics committee of the University of Ulm (4/2011, 312/2015, 19/2020) and ethics committee of the Ludwigs-Maximilians-University Munich (CORKUM, associated research project) and performed in accordance with the current Declaration of Helsinki. Each patient signed a letter of informed consent for the leave of blood and saliva samples.

### Biosampling

We established a biobank by collecting leftovers of blood samples from patients suffering from COVID-19 whenever sent to the central laboratory of our university hospital.

Blood for routine monitoring of the patients was normally withdrawn every Monday, Wednesday and Friday between 7:00 and 8:00 o’clock a.m. After measurement of routine parameters, the remaining serum was stored at -30°C until further measurements. Saliva sampling was performed by using the Salivette™ tube (Sarstedt, Nümbrecht, Germany).

### Kynurenine, IL-6 and CRP measurement

The method of kynurenine measurement was already published in detail (30). In short, serum samples were deproteinized with acetic acid trichloride, followed by consecutive proton-dominant hydrolysis. The stable metabolite kynurenine is reacting under the use of 4-dimethylamino-benzaldehyde (Ehrlich’s reagent) into a yellow product. Said coloring reagent serves for the detection of primary amino groups, pyrrole and indole derivatives as well. The colorimetric determination of the concentration is performed with monochromatic light. The standard solution of kynurenine was prepared by using L-kynurenine sulfate. Equal amounts of sample were mixed with 100 ml trichloroacetic acid (30%) thoroughly. Absorption was measured with 492 nm wavelength in a linear sector from 0.5–100 μM of the concentration of N-formylkynurenine, proportional to the activity of the enzyme indoleamine-2,3-dioxygenase. The absorbents of each sample at 492 nm were compared with the absorbents at 650 nm or 690 nm of the same sample. Then the absorbents of the controls were subtracted from the absorbents of each well. By preparing a standard curve the concentration of kynurenine in each sample could be determined.

Kynurenine concentration stability of stored samples for NC and the COVID-groups were observed (31). Thus, a reliable comparison of reference samples (NC) and the study groups A and B can be assumed.

IL-6 and CRP were measured with commercial immunoassays on a COBAS 8000 analyzer™ (Roche Diagnostics, Rotkreuz, Switzerland).

### Statistical analysis

Descriptive data analysis and analysis of variance methods were used to characterize the data. All p-values are two-sided and considered to be descriptive. For a formal statement of descriptive significance, a nominal type I error level of α=0.05 (two-sided) was assumed.

The exact Mann-Whitney U test was performed for comparison of two groups with not normally distributed continuous variables. Spearman’s rho (ρ) was performed to assess correlations between parameters of tryptophan metabolism and IL-6.

If not otherwise stated, all values are given as mean ± standard deviation. The analyses were performed using Sigma Plot 14™ from Systat Software Inc. (San Jose, CA, USA).

## Results

In this pilot-study, including n=85 patients infected with COVID-19 (mean age 52.8 ± 30.0 years) and n=302 NC, we first measured kynurenine in serum and subsequently in saliva (n=11).

### Serum kynurenine, IL-6 and CRP in group A and B on first admission

**Figure 2:**
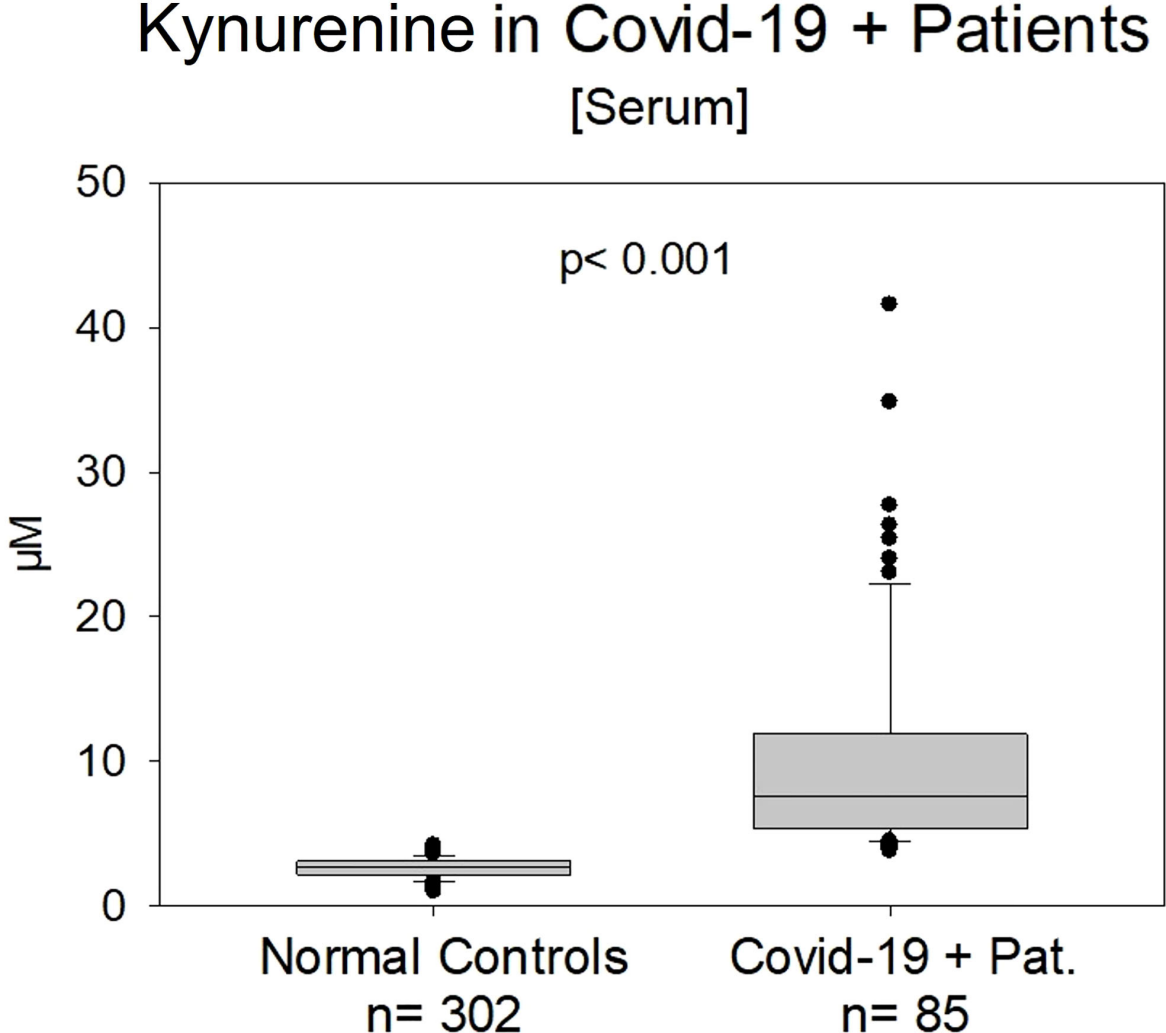
Kynurenine in normal controls (n=302) vs. COVID-19 patients in the early acute clinical state (n=85). The difference was significant (p< 0.001). We left out two results (58 and 43 µM) with an extreme hyperinflammatory syndrome in the COVID-19 positive group A.

On admission, serum kynurenine was significantly elevated in COVID-19 patients Group A compared to NC (10.81 ± 8.8 µM vs. 2.79 ± 0.61 µM; p<0.001) (**Figure 2**). Samples in Group A were taken by starting clinical treatment in the first week. For better graphical demonstration, we left out two results in the COVID-19 positive group (43 and 58 µM) with a hyperinflammatory syndrome.

Kynurenine significantly correlated positively with IL-6 peak-values (r=0.411; p<0.001) and CRP (r=0.488, p<0.001) (**Table 1**).

**Figure 3:**
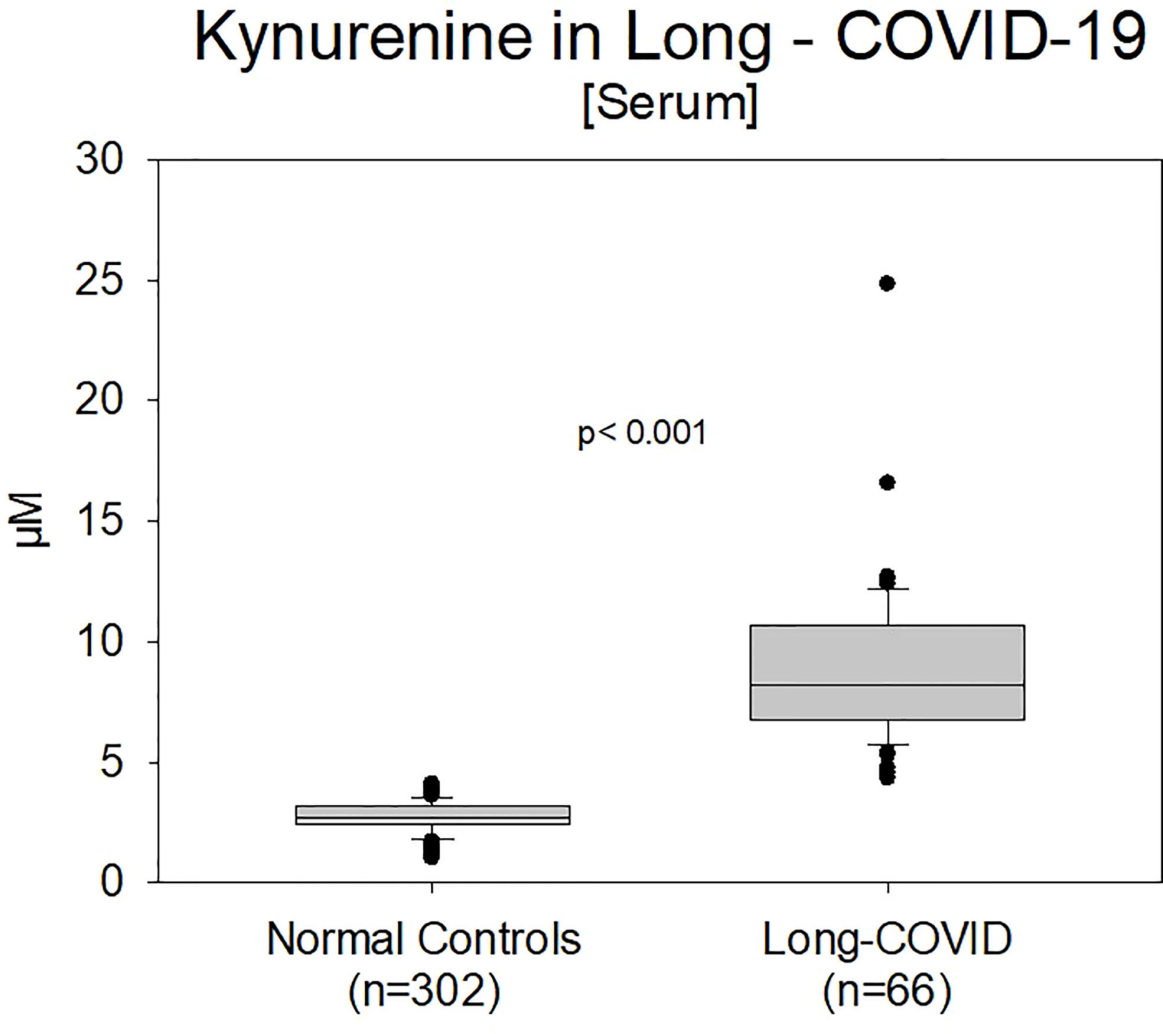
Comparison of kynurenine values between normal controls (n=302) and previously positive COVID-19 patients (Group B, n=66) with an existing Long-COVID syndrome (p<0.001). Patients with the LCS were currently under therapeutic management.

Kynurenine values in Group B (Long-COVID) showed still significantly higher values (8.77 ± 1.72 µM, range 5.5-24.6 µM, p<0.001 (**Figure 3**).

### Serum vs saliva kynurenine

**Figure 4:**
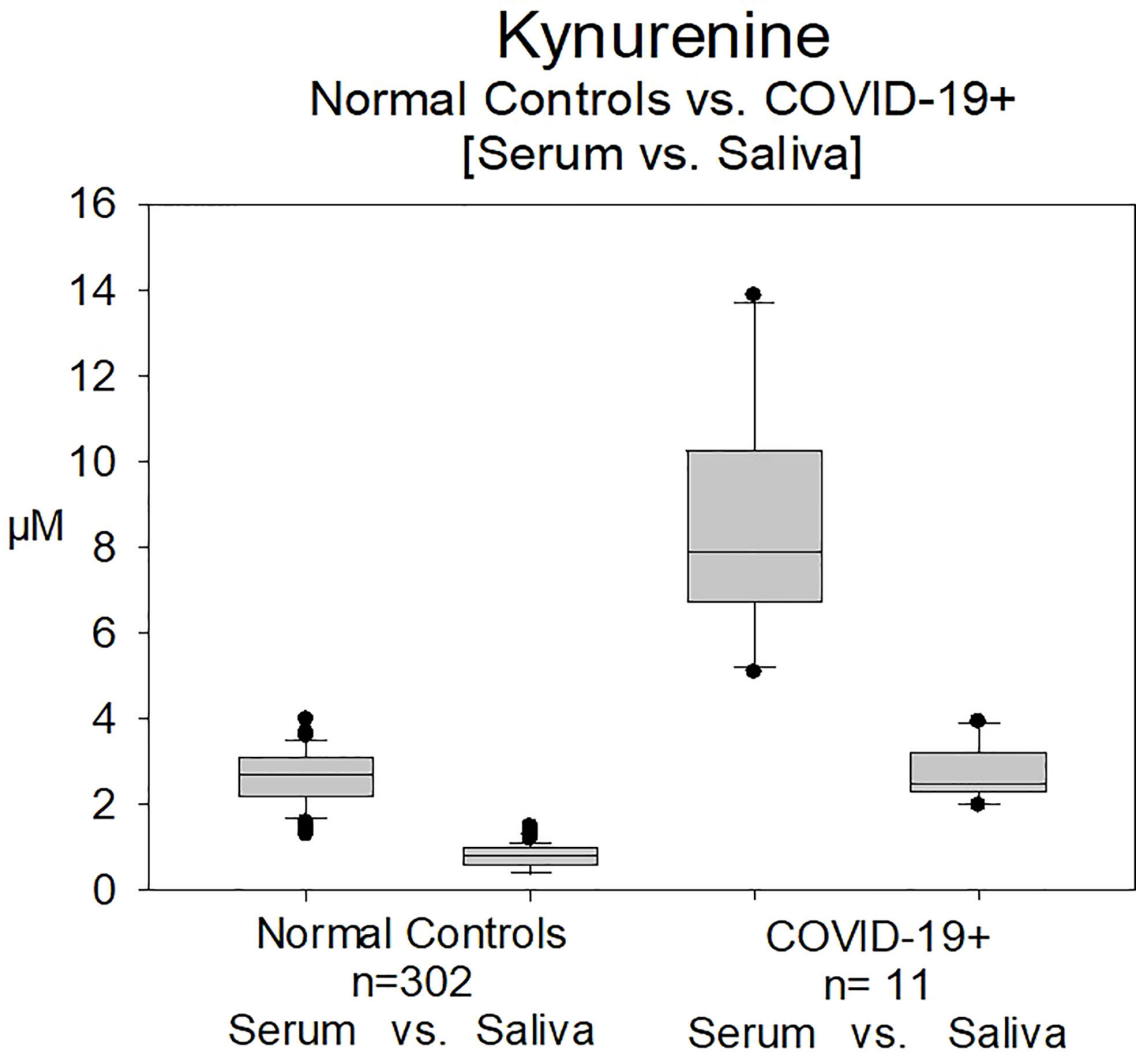
Measurement of Kynurenine in serum and in saliva in normal controls (n=302) and in patients with COVID-19 infection (Group B, n=11). Serum and saliva values in the previously COVID-19 positive patients were significantly higher compared to normal controls (p<0.001).

Kynurenine was measured not only in serum. In **Figure 4** the measurements of kynurenine of NC and COVID-19 positive patients (n=11) either measured in serum or saliva were compared according to the previous publication data of our group (30, 31). Serum and saliva values in the previously COVID-19 positive patients were significantly higher compared to NC (p<0.001).

### Serum kynurenine, IL-6 and CRP in group A and B on follow-up admission

**Figure 5:**
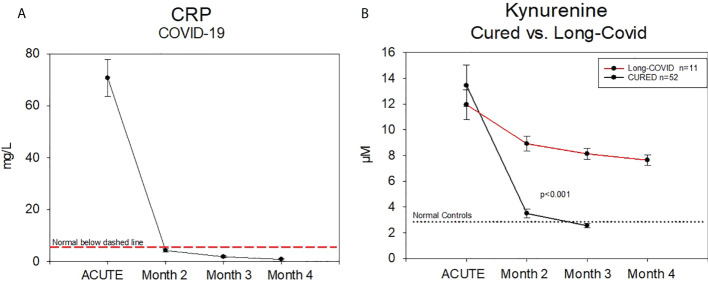
**(A)** Follow up of C–Reactive Protein and Kynurenine–measurement in patients either cured (n=11) or with a Long-COVID-syndrome (n=11) from a subset of Group B CRP was in a normal range after month 2 post infection (verified by a positive PCR-Test). **(B)** Kynurenine was still significantly increased.

Looking in the follow-up of a starting group of patients (n=11) with the diagnosis of Long-COVID syndrome, we could observe the sustained elevated level of kynurenine compared to NC from month 2 until month 4, the end of the follow up monitoring period so far. This was not found for CRP (**Figures 5A, B**). We compared 11 patients with positive infection and patients after COVID-19 positive infection without signs of a Long-COVID syndrome.

CRP, described in the literature as a good biomarker in COVID-19 patients (17), was not suitable to identify the Long-COVID syndrome. CRP at 2 months after positive testing was near the normal range of >5 mg/L or 5 µg/ml (**Figure 5A**). These findings are showing the difference between the Long-COVID (4-12 weeks) and Post-COVID syndrome (> 12 weeks).

**Figure 6:**
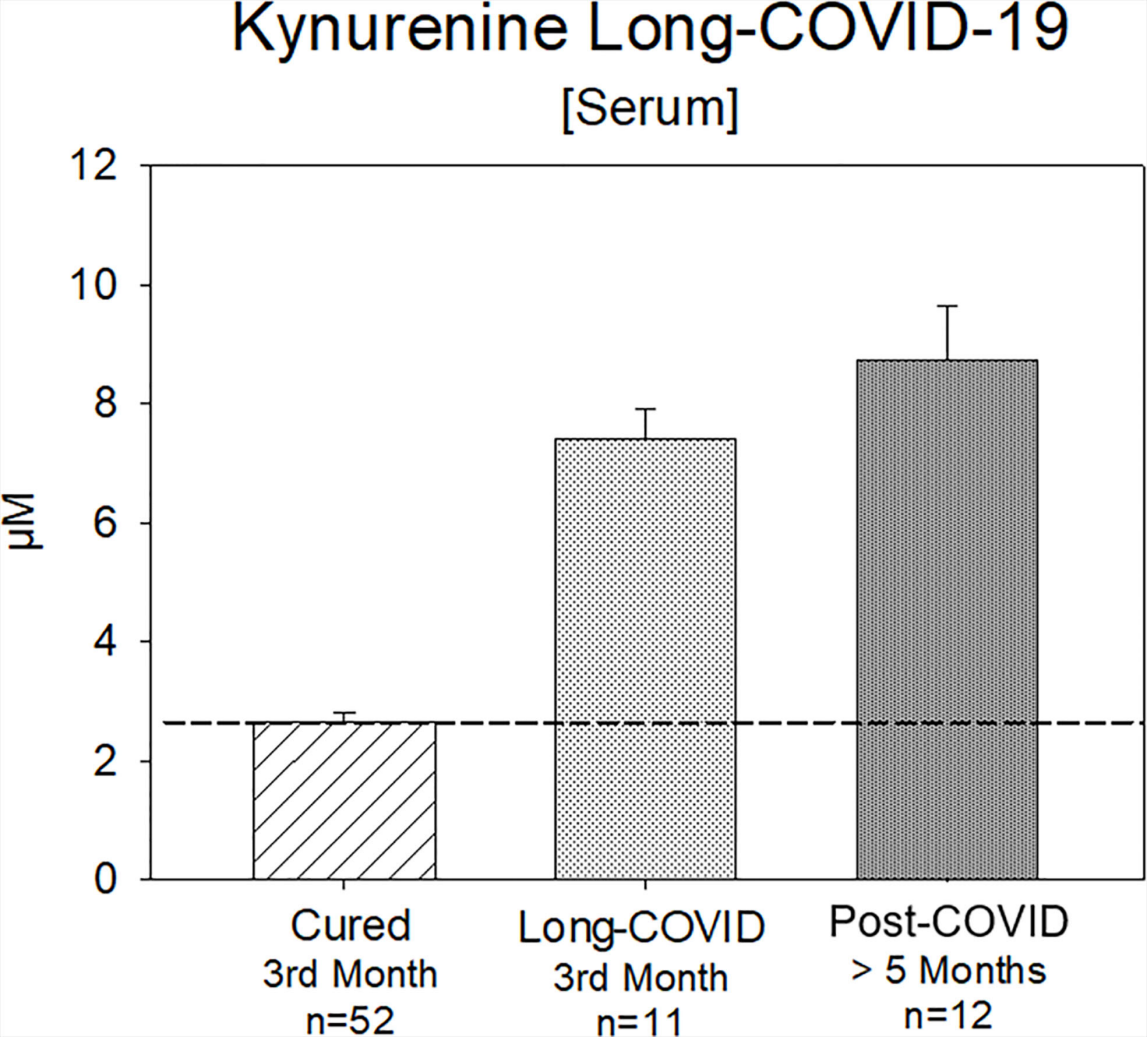
Kynurenine (Serum) in the 3rd month after positive PCR-testing: either cured or with a Long-COVID syndrome for 3 months or Post-COVID syndrome more than 5 months in a subset of Group B Kynurenine is still significantly increased, whereas the values of the cured patients are in a normal range.

12 patients with Post-COVID symptoms >20 weeks (range 20-42 weeks) with still elevated serum kynurenine-levels (8.73 ± 3.19 µM, range 5.4-16.5 µM) were identified (**Figure 6**).

## Discussion

Long-COVID is a chronic illness with a wide variety of symptoms, of which many are not explainable using conventional laboratory tests. There still are existing difficulties in detecting the illness. Researchers looking more deeply at Long-COVID patients have found visible dysfunctions throughout the body (22, 32).

Although it was shown that the marker CRP might be helpful during the acute phase of the disease (16, 17), the long-term value remains questionable. Our data underline the appropriate use of CRP in the acute phase, while CRP concentrations in Long-COVID are comparable with normal controls and thus do not reflect the long-term inflammation. This was supported by previous results of our group in renal transplanted patients, where the diagnosis of rejection could not be estimated by CRP-monitoring (30).

In contrast, kynurenine was still increased up to four months in patients suffering from Long- and Post-COVID compared to cured COVID-patients without developing Long-/Post-COVID (subpopulation of Group B) or NC. In addition, the picture of hyperinflammation with extraordinary high levels of kynurenine was solely found in COVID-19 patients, especially in those on the ICU with kynurenine concentrations up to 79.4 µM (data not shown).

Furthermore, the accumulative evidence of the COVID-19 specific studies published in the last year suggests that SARS-CoV-2 infection induces a powerful, and apparently uncontrolled inflammatory response. This inflammation can be assumed to contribute to the tissue damage already caused by the viral infection towards the COVID-19 underlying pathology. The short- and long-term sequalae following recovery of COVID-19 suggests that these syndromes lead to an accelerated state of chronic subclinical systemic inflammation. Thus, a reliable immunological marker to support prognosis in the acute and long-term phase of the COVID-19 disease and which reflects the current inflammatory status would be a valuable asset.

Studies estimate that around 10-30% of people infected with SARS-CoV-2 may develop long-term symptoms. Four different risk factors were identified for a more severe development: a) the presence of having Type 2 diabetes, b) reactivation of Epstein-Barr virus (EBV) c) presence of certain autoantibodies and d) high levels of viral RNA early during an infection (16, 24).

The interdependence of virus infection with kynurenine and the activation of the kynurenine pathway in SARS-CoV-2 positive patients has been examined by fellow working groups. Lawler et al. evaluated indicators of the tryptophan metabolism by quantitative metabolic phenotyping and found that especially the neurotoxic metabolites kynurenine, quinolinic acid and 3-hydroxykynurenine were increased in ten SARS-CoV-2 positive subjects (33). This finding was confirmed by the metabolomic study of Thomas et al., who observed a profound alteration of the kynurenine pathway in patients with SARS-CoV-2 infection (34). In their study population of 33 SARS-CoV-2 positive subjects, a significant decrease of tryptophan with concomitant increases of kynurenine, kynurenic acid and picolinic acid was observed.

Furthermore, the association between the disease outcome and the plasma levels of kynurenine pathway metabolites demonstrates that indicators of tryptophan metabolism - especially but not limited to kynurenine - may have the potential as prognostic biomarkers in individuals with SARS-CoV-2 infection. Kynurenine levels of patients compromised by virus-infections (Cytomegalovirus vs. COVID-19) are comparable, but much more pronounced in COVID-19 (30). In addition, we already showed that kynurenine is elevated in patients suffering from overtraining symptom and associated chronic fatigue (35), which is also a common side effect of Long- and Post Covid, compared to healthy individuals.

Post-COVID patients appear to have a disrupted immune system to Long-COVID patients who fully recover. One reason is that the body is still fighting remnants of SARS-CoV-2. Other groups found that the virus spreads widely during an initial infection, while SARS-CoV-2 specific genetic remnants can remain in tissues for many months (i.a., in the intestines and lymph nodes) (36). A further possibility is that the initial viral infection induces chronic inflammatory processes, possibly by reactivating other viruses in the patient’s body that are normally dormant. Most individuals are infected during their childhood and adolescence, and its reactivation might help predict whether a person will develop Long-COVID.

Beside CRP, we found correlations between kynurenine and peak IL-6 values in the acute infection phase. Long-term assessments are still undergoing, but recent studies point to a link between Long-COVID and elevated IL-6 concentrations (37). Hence, the simultaneous determination of EBV, IL-6 and kynurenine may therefore complement each other in a “Long-COVID-Panel” for the clinical assessment and monitoring, as all three can reliably be measured non-invasive in saliva (38), and might provide even higher prognosis accuracy.

Thus, our results are demonstrating the relevance concerning diagnosis and monitoring of a Long- and Post-COVID syndrome and PIMS in children and the potential for other virus induced inflammation monitoring. Furthermore, recent data indicates that the maintenance of gut dysbiosis and increased gut permeability, evident in acute COVID patients, may underpin Long-COVID (39). Given that alterations in gut dysbiosis/permeability impact on systemic mitochondrial function, including in immune and glial cells (40) as well as increasing the pro-inflammatory cytokines driving IDO and the conversion of tryptophan to kynurenine, the role of gut dysbiosis/permeability interactions with kynurenine, serotonin and melatonin may be important to examine. It should also be noted that the kynurenine activation of the AhR will actively suppress natural killer cells, and therefore the capacity of the body to detect and eliminate virus-infected cells as well as cancer cells (41).

Above that, our pilot experiment of determining kynurenine in serum and subsequently in saliva demonstrates the additional usefulness of clinical kynurenine monitoring. The non-invasive kynurenine measurement in saliva is a safe and fast approach of assessing the disease status of COVID-19 patients that is not biased by blood-thinning medications or other therapy targeting the cardiovascular or blood system.

Taken together, our data supports the following possible theory behind the Long-COVID syndrome: the longer lasting activation of the innate immunity, triggered additionally by genetic material of the virus in tissue, induce DAMP-like inflammatory reactions, which may end up in a subclinical chronic inflammation. This subclinical chronic inflammation could be detected by measuring kynurenine that opens a window for new therapeutical approaches which could be easy monitored.

### Strengths and limitations

Nevertheless, some limitations of this study must be considered. Due to the retrospective study design, only the routinely measured parameters were available for statistical analysis. Furthermore, kynurenine concentrations in saliva and serum of Long- and Post-COVID patients were only determined in a limited subsample of the whole cohort, which limits the statistical effect size.

A strength of the study is that none of the patients in Group A or B was under hemodialysis. Therefore, an influence on the kynurenine level by this treatment is unlikely. Above that, this pilot study was partly designed as a “proof-of-concept” study to show the prognostic value of kynurenine monitoring. Although the preliminary results are promising, higher participant numbers are needed for still higher validity and reliability of the presented data.

Another source of bias may be the parenteral substitution of amino acids in ICU patients. We do not have information about the exact nutritional program of the included ICU patients. Hence, we cannot rule out an influence on the measurements of the tryptophan metabolites, although the effect might be supposedly weak (42). Considering a normal diet, the daily uptake is nearly five times higher than the need. Lack of supply for tryptophan is only existent in heavenly starving people and not in the western hemisphere, so an influence on the kynurenine level is again unlikely. Additionally there are a few more mechanisms regulating the tryptophan pathway (43) which need to be considered in interpreting the observed results.

### Conclusion and future perspectives

Kynurenine can be regarded as a useful biomarker in detection of the inflammatory and hyperinflammatory character of the SARS-CoV-2 disease in the acute as well as the long-term progression. Furthermore, kynurenine is able to detect the chronic subclinical systemic inflammation typical for the Long-COVID- and more pronounced for Post-COVID syndrome.

In addition, we could demonstrate in a sub study with pilot character that in a subpopulation of previously COVID-19 positive patients, kynurenine could be the first time measured additionally in saliva and serum with comparable results. We are working on a test (ELISA as well as a LFA-format) to translate this biomarker testing in a new format either concerning the matrix (saliva) and the technique. Measurements in saliva opens the opportunity for self-monitoring of the patients and noninvasive therapy control.

Given the wider alterations arising from the conversion of tryptophan to kynurenine, including suppressed levels of serotonergic and melatonergic pathway activation (44), it will be important to determine the relevance of such coordinated changes together with kynurenine monitoring in acute, Long- and Post-COVID. Further pathway studies including the different sides of kynurenine pathways (hepatocytes, muscle cells, gut lumen) may shed additional light on the involvement of kynurinen and its metabolites in virus driven disease like COVID-19 and assist in clinical monitoring and disease outcome. Investigations on such processes will allow for better integration of the role of raised kynurenine levels in both Acute and Long-COVID.

## Data availability statement

The original contributions presented in the study are included in the article/supplementary material. Further inquiries can be directed to the corresponding author.

## Ethics statement

The studies involving human participants were reviewed and approved by ethics committee of the University of Ulm (4/2011, 312/2015, 19/2020) and ethics committee of the Ludwigs-Maximilians-University Munich (CORKUM, associated research project). The patients/participants provided their written informed consent to participate in this study.

## Author contributions

DB, MS, DA, and J-LB contributed to conception and design of the study. DA and MS organized the database. DB, MS, JD, GD, J-LB, and DA performed the statistical analysis. DB, MS, and DA wrote the first draft of the manuscript. JD and GD wrote sections of the manuscript. All authors contributed to the article and approved the submitted version.

## Acknowledgments

We gratefully acknowledge the continuous support of Prof. Dr. Jürgen M. Steinacker (Head of the Division of Sports and Rehabilitation Medicine Ulm) by allowing to use his laboratory and his intellectual input as well as B.Sc. Lucas John for the experimental analysis. We thank our collaboration partners JC Hellmuth (Department of Medicine III, University Hospital Munich, Bavaria, Germany), Clemens Scherer (Department of Medicine I, University Hospital Munich, Munich, Bavaria, Germany) and Maximilian Muenchhoff (Virology, Ludwig Maximilians University of Munich, Munich, Bavaria, Germany).

## Conflict of interest

The authors declare that the research was conducted in the absence of any commercial or financial relationships that could be construed as a potential conflict of interest.

## Publisher’s note

All claims expressed in this article are solely those of the authors and do not necessarily represent those of their affiliated organizations, or those of the publisher, the editors and the reviewers. Any product that may be evaluated in this article, or claim that may be made by its manufacturer, is not guaranteed or endorsed by the publisher.
